# The Relevance of Oxidative Stress in the Pathogenesis and Therapy of Retinal Dystrophies

**DOI:** 10.3390/antiox9040347

**Published:** 2020-04-23

**Authors:** Elena B. Domènech, Gemma Marfany

**Affiliations:** 1 Departament de Genètica, Microbiologia i Estadística, Avda. Diagonal 643, Universitat de Barcelona, 08028 Barcelona, Spain; elenabdomenech@ub.edu; 2 CIBERER, ISCIII, Universitat de Barcelona, 08028 Barcelona, Spain; 3 Institute of Biomedicine (IBUB, IBUB-IRSJD), Universitat de Barcelona, 08028 Barcelona, Spain

**Keywords:** oxidative stress damage, oxidative stress response, photoreceptors, retina, age-related macular degeneration (AMD), glaucoma, retinitis pigmentosa, leber hereditary optic neuropathy (LHON)

## Abstract

Retinal cell survival requires an equilibrium between oxygen, reactive oxygen species, and antioxidant molecules that counteract oxidative stress damage. Oxidative stress alters cell homeostasis and elicits a protective cell response, which is most relevant in photoreceptors and retinal ganglion cells, neurons with a high metabolic rate that are continuously subject to light/oxidative stress insults. We analyze how the alteration of cellular endogenous pathways for protection against oxidative stress leads to retinal dysfunction in prevalent (age-related macular degeneration, glaucoma) as well as in rare genetic visual disorders (Retinitis pigmentosa, Leber hereditary optic neuropathy). We also highlight some of the key molecular actors and discuss potential therapies using antioxidants agents, modulators of gene expression and inducers of cytoprotective signaling pathways to treat damaging oxidative stress effects and ameliorate severe phenotypic symptoms in multifactorial and rare retinal dystrophies.

## 1. Introduction

Oxidative stress (OS) alters cellular homeostasis and elicits a cell response that depends on the severity and the type of insult. Essentially, there is a limiting stress threshold: below the threshold, cells elicit protective mechanisms designed to ensure survival. Conversely, if stress surpasses the threshold or the activation of protective mechanisms fail, cells trigger alternative signaling pathways that eventually lead to apoptosis, necrosis, pyroptosis, or autophagic cell death [1,2,3,4].

Cell survival requires an equilibrium between oxygen and antioxidant molecules. Reactive oxygen species (ROS) such as superoxide anion (O_2_^•−^), oxygen peroxide (H_2_O_2_), hydroxyl radical (OH^•^), peroxy radical, and the second messenger nitric oxide (NO^•^) (which interacts with superoxide anion to form peroxynitrate (ONOO^−^)), are the major sort of ROS present at the cellular level. Usually, there is an equilibrium between oxidative species and antioxidant defense mechanisms, which are mediated by enzymes responsible for metabolizing or neutralizing ROS, e.g., catalase, glutathione peroxidase, or superoxide dismutase (SODs) [5,6,7,8].

ROS are generated in many enzymatic processes and in redox reactions. Mitochondrial respiratory chain is one of the main ROS sources in cells. In the inner mitochondrial membrane, electrons are transported and oxygen is converted into water. Under hypoxia conditions this process is not completed, resulting in an increase of superoxide anions. The nicotinamide adenine dinucleotide phosphate oxidase (NOX) is the main source of ROS derived from superoxide anions. NOX is an oxidase family of seven members, and among them is NOX4, which generates superoxide anions and hydrogen peroxide [7].

**Figure 1:**
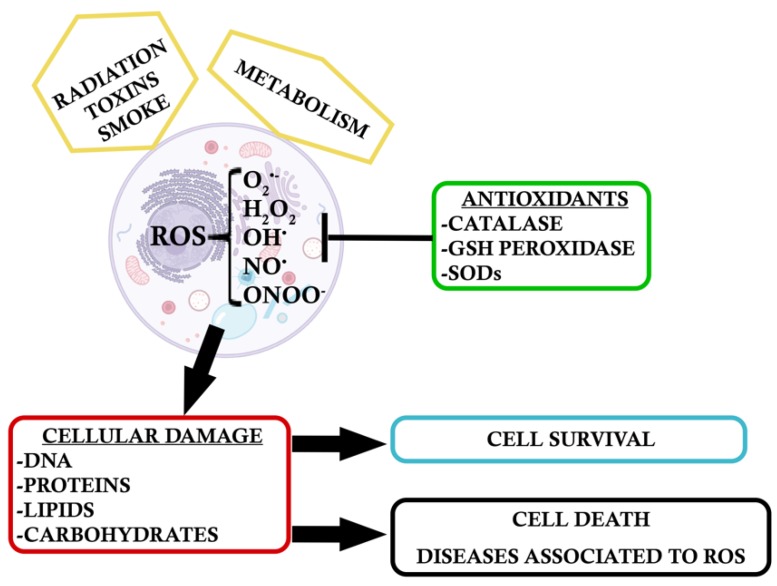
Oxidative stress agents generate Reactive Oxygen Species (ROS), which are counteracted by antioxidant enzymes. An excess of oxidative stress leads to cellular damage, which can trigger pathways for either cell survival or cell death.

These free radicals are small molecules/ions that are reactive with small activation energies and short lifetimes [9]. There are many environmental agents that can promote ROS generation (e.g., pollution, cigarette smoke, sunlight). Nonetheless, they can also result from reactions occurring in the body like incomplete catabolism, hepatic detoxification or energy production. An intracellular increase of these oxidant agents causes damage to different cellular components and as a consequence, specific pathways linked to cell senescence are activated [10]. OS can damage lipids, proteins enzymes, carbohydrates, and DNA, thus inducing cell death (Figure 1). In humans, OS is at the basis of several neurodegenerative and cardiovascular diseases, cancer, diabetes, and autoimmune disorders [4,10], but the focus of this review is the oxidative stress damage in the retina and the diseases associated to an impaired cell response.

## 2. Environmental Oxidative Stress

### 2.1. Air Pollution and Cigarrete Smoke

Air pollution and cigarette smoke are environmental toxic factors that induce the production of ROS. Exposure of the eye to air pollutants increase the risk of suffering glaucoma due to the increase of ROS and the consequent inflammatory response [11]. Cigarette smoke contains nicotine, which promotes nitric oxide production, a molecule that increases the levels of proangiogenic factors [12], whereas the cadmium present in tobacco accumulates into retinal pigment epithelium (RPE) cells and the choroid, thereby increasing ROS production [13]. Additionally, hydroquinone (HQ), a natural oxidant present in food, plastics, and polluted air, is also induced by cigarette smoke. Finally, several studies show that cigarette smoke extract (CSE) induces alterations in mitochondrial integrity and an increase of lipid peroxidation, thus leading to cell death in the retinal pigment epithelium (RPE) [14].

### 2.2. Light-Induced Oxidative Stress

Humans are essentially diurnal animals and are constantly exposed to sunlight, which induces lipid and protein oxidation. In fact, mammalian retinas are especially sensitive to light (which also include high energy UV wavelengths) stress. Photoreceptors and RPE cells generate high levels of ROS due to different factors: (i) the retina is a neuronal tissue characterized by extremely high oxygen consumption related to a highly demanding metabolism; (ii) photoreceptors are constantly exposed to light and contain several photosensitizer molecules; (iii) photoreceptor membranous disks are enriched in polyunsaturated fatty acids, which are particularly sensitive to oxidation damage; and (iv) the daily recycling of photoreceptor disks damaged by oxidative stress is performed via phagocytosis by RPE cells, and consequently, oxidative damage to photoreceptors induces accumulation of ROS in the RPE. Photooxidation causes lipofuscin accumulation, which is the oxidation byproduct of lipids and lipoproteins containing photo-oxidable fluorophores [15,16]. High levels of light-induced oxidative stress eventually triggers photoreceptor and inner retina apoptosis [17,18] and severely affects the phagocytic function of the RPE [19].

## 3. Intrinsic Response to Oxidative Stress

### 3.1. Inflammation and Gliosis

An inflammatory response is a natural defense mechanism against injuries. Innate immunity is considered the immediate immune response to an insult or pathogen. The retina is equipped with a highly sensitive innate immune system that responds with three key pathways: migration of microglia cells, activation of the complement system to opsonize cellular debris, and inflammasome assembly in the RPE (reviewed in [20]). To achieve this coordinated response, retinal cells express a panoply of immune protein receptors and mediators such as, microbial sensors (Toll-like receptors-TLRs), NOD-like receptors-NLRs, RIG-1 like helicases), cytokines, chemokines, as well as a group of complement components; all directed to assist the cells with eliminating the current insult [21]. In oxidative stress conditions, rapid activation of this immune response is intended to induce restoration of tissue homeostasis, but upon persistent damage, chronic overactivation of the inflammatory response can cause devastating tissue remodeling and destruction, thus leading to irreversible retinal pathologies, such as age-related macular degeneration (AMD) or diabetic retinopathy (DR).

Several chronic diseases are associated with protein oxidation due to high ROS production. Protein oxidation activates the release of inflammation regulatory proteins such as peroxiredoxin 2 (PRDX2). The role of OS as a key trigger of pathogenic inflammation in chronic inflammatory diseases has been well established. Indeed, pro-inflammatory cytokines such as TNF-α, interleukin-1β, or interferon-γ induce ROS production in RPE cells. In fact, these pro-inflammatory cytokines are upregulated in the eyes of patients suffering glaucoma, age-related macular degeneration (AMD), diabetic retinopathy, or retinal vein occlusion. Particularly, endothelial cells affected by inflammation show phenotypic changes due to the increase of the expression of inflammatory mediators and cytokines as well as iNOS activation [21].

As mentioned, one of the first response of the retina to external damaging stimulus, such as oxidative stress, is reactive gliosis, in which astrocytes, microglial, and macroglial cells are activated. Microglia cells are resident retinal macrophages that confer neuroprotection against ROS damage and other injuries. In healthy retinas, microglial cells phagocyte accumulated waste products and cellular debris but under oxidative stress, hypoxia, or inherited mutations, NFκB-mediated inflammatory responses are activated, and microglial cells acquire amoeboid morphology, proliferate, and migrate to the sites of injury, as deteted by Iba-1 positive cells in the photoreceptor layer [21,22]. Oxidative stress promotes the degradation of sialic acid residues of membrane proteins, leaving photoreceptors and other cells with a damaged glycocalyx, and leading to enhanced phagocytosis by microglial cells, and increasing neuronal cell death, which worsens the pathology.

On the other hand, macroglia Müller cells, which constitute the columns of the retinal tissue and with multiple connections with retinal neurons, microglia, astrocytes, and endothelial cells, modulate different responses according to the severity of the stimulus. The activation of these macroglial cells involves hypertrophy, which entails overexpression of vimentin (an intermediate filament) and the glial-acidic fibrillary protein (GFAP), considered a hallmark of retinal stress. As an immediate response to acute non-permanent stimuli, Müller cells promote the secretion of trophic and antioxidant protective factors, but upon chronification, their secretory role may be clearly deleterious for neuronal cells [23].

### 3.2. Endoplasmic Reticulum Stress

Endoplasmic reticulum (ER) is the main cellular compartment responsible for protein biosynthesis and folding. This organelle is one of the main cell sensors to stress insults. Alterations of the ER redox status can negatively affect protein folding and result in ER stress [24]. ER stress leads to the activation of three transmembrane proteins: inositol-requiring enzyme 1α (IRE1α), PKR-like ER kinase (PERK), and the activation transcription factor 6 (ATF6), which in turn or jointly initiate the unfolded protein response (UPR) [25,26]. Several reports show the role of UPR in controlling OS response and cell survival in RPE cells [27,28].

The retina and RPE of mice exposed to cigarette smoke showed an increase of CHOP, a sensor that triggers cell apoptosis. In cultured human RPE cells, inhibition of ER stress attenuated apoptosis and cell death induced by oxidants including hydroquinone and CSE [28,29]. Usually, transient ER stress can be overcome by UPR but if the damaging stimulus persists, inflammatory response genes are activated [25,26]. Indeed, the accumulation of misfolded proteins plays a key role in the development and progression of ocular diseases such as retinitis pigmentosa (RP), AMD, and glaucoma [25].

On the other hand, intrinsic genetic factors, such as several causative dominant mutations in RP, cause protein misfolding of highly expressed transmembrane proteins, which causes ER stress and activation of the unfolded protein response (UPR) in photoreceptors cells. Chronically activated UPR in degenerating retinas activate pro-apoptotic programs associated with oxidative stress, pro-inflammatory signaling, dysfunctional autophagy, free cytosolic Ca^2+^ overload, and altered protein synthesis rate in the retina (as extensively reviewed in [30]).

### 3.3. High Metabolic Rate of the Retina

Photoreceptor outer segments are densely packed with membranes and opsin proteins [31]. In fact, lipids constitute 15% of the mass of a photoreceptor. Photoreceptors need large amounts of ATP to maintain the membrane potential [32], as the outer segments of photoreceptors are in constant renewal. Mutations in genes that compromise cell metabolism, such as the *isocitrate dehydrogenase-3β* gene (which encodes a mitochondrial enzyme), are associated with photoreceptor degeneration and result in RP [33].

The isomerization of 11-*cis*-retinal in the visual cycle can lead to the formation of compounds that are reactive with short wavelength light, generating free radicals [34]. The high metabolic rate of photoreceptor cells is an intrinsic factor that causes ROS as a consequence of the high mitochondrial metabolism. Moreover, photoreceptor cells are localized near arteries due to their high demand of oxygen, but upon photoreceptor death, consumption in the retina decreases. Blood circulation in the choroidal vessels does not adapt to this decrease and thus, the consequent hyperoxia could cause oxidative damage [35].

On the other hand, mTOR levels or activity might be altered in some inherited visual disorders. The activity of mTOR, a key regulator of cellular metabolism (e.g., translation, macroautophagy, and metabolic pathways), is regulated by phosphorylation. Several studies showed that the phosphorylation of mTOR was reduced in the dorsal cones of retinitis pigmentosa mouse models. In addition, L/M opsin protein levels were also decreased, probably reflecting a reduction in translation, which is under the control of mTOR. Thus, mTOR phosphorylation status in RP might be low due to the cells suffering from some type of nutrient deprivation and/or metabolic dysregulation [32], further linking metabolism alterations and oxidative stress to retinal pathogenesis (see below, Section 10).

## 4. Endogenous Antioxidants: Dual Role of NF-κB and the Antioxidant NRF2

Although ROS are toxic agents for cell viability, particularly in chronic conditions, they can also play a beneficious role when present at low levels because these molecules activate cell protective mechanisms upon injury.

The balance between oxidants/antioxidants is finely regulated to avoid ROS to overcome a damaging threshold. Cells enable different mechanisms to counter the OS with antioxidants, which can be produced endogenously in an endocrine or paracrine pathway, or exogenously, acquired from food or supplements. In either case, antioxidants work as scavengers to neutralize free radicals, preventing and repairing the damage caused by ROS, thus antioxidants enhance immune defense and reduce the risk of suffering some diseases.

Antioxidant enzymes play a key role in the first response to reduce ROS levels, hence redox regulation by transcription factors is essential in determining gene expression profiles and coordinating cell response under oxidative stress conditions [36]. Cells are equipped with several kinds of antioxidants: (i) low-molecular-weight antioxidants, such as ferritin, ascorbic acid, reduced glutathione (GSH), and α-tocopherol, etc., and (ii) high-molecular-weight antioxidants, such as catalase, superoxide dismutase, glutathione peroxidase and reductase, etc. [37]. In this review, we will focus on two essential antioxidant pathways essential to battle oxidative stress, controlled by specific transcription factors, NF-κB and NRF2.

The nuclear transcription factor κB (NF-κB) belongs to a transcription factor family involved in inflammatory and immunity pathways. NF-κB also plays an important role in developmental processes, cell growth and survival, proliferation and several pathological conditions. NF-κB plays a dual role upon oxidative stress: despite in most cases works as an antioxidant agent promoting cell survival, it may also function as an oxidant agent and causes cell death. NF-κB influence ROS levels increasing antioxidant proteins expression (among others, SODs, ferritin, thioredoxin, peroxidase glutathione, dihydrol dehydrogenase), whose function is to protect cells against oxidative stress by reducing or neutralizing ROS. On the other hand, NF-κB plays an important role in activating inflammatory pathways because it also targets genes encoding enzymes that trigger ROS production (NOX2, iNOS, nNOS, cyclooxygenase 2, etc.), and their increase in activity can eventually lead to cell death [38,39].

The nuclear factor- erythroid 2-related factor 2 (NRF2, also named NFE2L2) is a transcription factor (TF) that regulates antioxidant defenses. In normal conditions, NRF2 is lowly expressed and localizes at the cytoplasm, where it interacts with KEAP1, which favors its ubiquitination and degradation via proteasome. In contrast, under oxidative stress conditions, NRF2 expression levels increase and it is translocated to the nucleus, where dimerizes with MAF proteins and binds to gene promoters that encode antioxidant enzymes such as SODs, catalase, S-transferase glutathione, which in combination, scavenge ROS [4,7,8].

NRF2- and NF-κB-pathways regulate the fine redox cellular balance, oxidative stress, and the inflammatory response. Imbalance between NRF2 and NF-κB pathways is associated with a significant number of diseases ranging from neurodegeneration and autoimmune disorders to cancer. The interaction between these two pathways is carried through complex molecular interactions that usually depend on the cell type and the tissue context. These interactions act through transcriptional and post-transcriptional mechanisms, and allow finely tuned dynamic responses to a changing environment [39,40]. Therefore, it comes as no surprise that they are also relevant in retinal pathologies associated to oxidative stress, for instance in AMD (see below, Section 9).

## 5. Stress Granules (SG) Formation

In response to environmental stress (heat, hyperosmolarity, oxidative stress, etc.) eukaryotic cells shut down protein synthesis in a stereotypic manner to preserve anabolic energy to repair stress induced damage. The translational arrest is selective: while the translation of constitutively expressed genes (housekeeping genes) is hampered, the translation of stress-inducible transcripts encoding heat shock proteins among others is enhanced or favored [41]. To protect those mRNAs that are not going to be translated yet, the cell initiates a signaling cascade to form stress granules (SG), which are a complex of a variable number of proteins that bind and protect mRNAs upon stress stimuli in order to avoid their degradation.

On top of the signaling cascades responsible for cell translation reprogramming upon stress are the family of serine/threonine kinases. Some members of this family of environmental stress sensors are: (i) PKR, a double strand RNA-dependent kinase, activated with viral infections, heat, and UV radiation; (ii) PERK/PEK, an ER protein that is active in response to the accumulation of misfolded proteins in the ER; (iii) GCN2, a sensor of intracellular amino acid levels that responds to amino acid deprivation; (iv) HRI, a hemo-regulated kinase that ensures the synthesis of globin chains and hemo during erythrocytes maturation. All of these sensors phosphorylate eIF2α.

**Figure 2:**
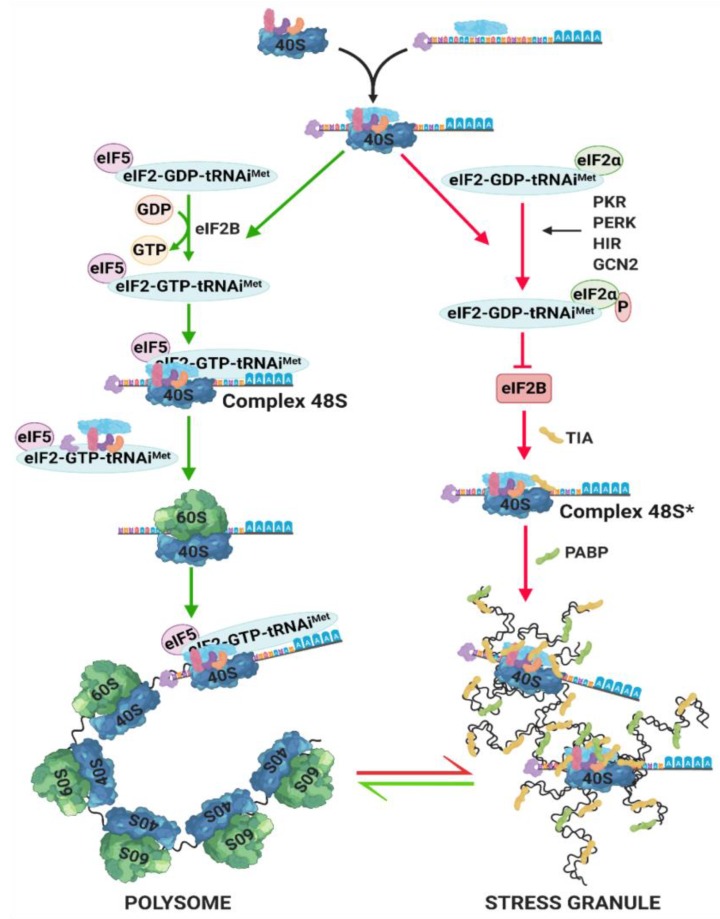
Translational start in presence or absence of cellular stress. In normal conditions and in absence of stress (green arrow pathway), eIF2B catalyzes the exchange of GDP to GTP and allows the coupling of the ternary complex to the 48S complex. The ribosomal subunit 60S can join the complex and the addition of more ribosomes to this complex make a polysome, which is able to translate the mRNA. Upon cellular stress (red arrow pathway), eIF2α is phosphorylated by stress sensor kinases and inhibits eIF2B, precluding the binding to EIF5. The ternary complex generates a 48S* complex instead, which is deficient in eIF2/eIF5 and unable to start the translation. This complex recruits TIA-1 and TIAR proteins to form the SGs. Additionally, SGs recruit more RNA binding proteins, such as PABP. mRNAs move in and out of SGs depending on the cellular translational requirements. Thus, SGs are highly dynamic complexes, in equilibrium with polysomes.

eIF2B is a GTP/GDP exchange factor and a key regulator of the ternary complex (composed by the unit eIF2αβγ, a tRNAi^Met^, and GTP), which loads the initiator tRNAi^Met^ on the small ribosomal subunit to start protein synthesis. Translation is usually started when eIF5 hydrolyzes GTP in the pre-initiator complex and its initiation factors are moved out allowing the big ribosomal subunit to couple. The additional ribosomes coupling turns the RNA into a polysome (Figure 2). Upon oxidative stress, the activation of one or more eIF2α kinases results in the phosphorylation and consequent inhibition of eIF2α, thus reducing the availability of the eIF2-GTP-tRNAi^Met^ complex, and the binding to EIF5 [41] (Figure 2). Pre-initiator complexes deficient in eIF2/eIF5 stall the complex, which is then named 48S* [42]. The 48S* complexes and their cognate mRNAs are redirected in a process that requires the RNA binding proteins, TIA-1 and TIAR. These two proteins, usually localized into the nucleus, are highly dynamic and shuttle from the nucleus to the cytoplasm. In response to cellular stress, TIA-1 and TIAR accumulate into the cytoplasm, bind the 48S* complexes and rapidly promote the formation of SGs [41] (Figure 2). SGs are membrane-less organelles that behave as lipid droplets [43]. The absence of the big ribosomal subunit within these aggregates precludes SGs being translational foci during the duration of the stress stimulus. RNA binding proteins, like PABP, could either promote or inhibit mRNA stability and are also recruited to SGs, in consequence the mRNA fate is determined by the activity of these proteins. However, the presence of the small ribosomal subunit within these aggregates indicates that SGs could be related with polysomes since the latter show a very similar composition to SGs. SGs are highly dynamic complexes and the fact that the mRNAs shift in and out of the SGs leading to a dynamic equilibrium between polysomes and SGs (Figure 2) [41,42,44].

Recently, the insoluble transcriptome of RNA binding proteins (RNPs) has been characterized in response to different stress stimulus, for instance, in response to ER stress, oxidative stress induced by a toxic agent like sodium arsenite, or heat shock. These analyses show that there is a dynamic and differential distribution of RNAs in the soluble fraction of the cytoplasm and cytosolic RNPs depending on the stress stimulus [45]. Therefore, the differential recruitment of distinct RNAs into SGs could be one of the main cell regulatory mechanisms of gene expression and the functional transcriptome as a response to stress. Different RNAs will be protected depending on the stress stimuli, tissue and cell requirements. Defective regulation of the subcellular localization of RNAs or mutations in the proteins involved in the formation of the RNPs and SGs are associated to several human diseases including cancer and neurodegeneration [45,46]. In these diseases, in which cells are usually under continuous stress, the dynamic relationship of RNA and proteins in response to shifting extreme stress conditions could be a key factor to understand their etiopathogenesis.

Again, neuronal tissues, such as the retina, need a careful balance between the response to oxidative stress and the formation of SGs; between stalling translation and producing the protein levels physiologically required for cell survival.

## 6. Oxidative Stress Impact on Autophagy and Mitophagy

### 6.1. Autophagy and Oxidative Stress

Autophagy is a conserved cell survival pathway that catabolizes damaged proteins and organelles to maintain homeostasis. Nonetheless, in response to different stimuli and cellular contexts, autophagy can also modulate cell viability, either enhancing cell survival or cell death. Autophagy is involved in a number of different processes in the retina participating in development and tissue remodeling and it is also implicated in photoreceptor cell death in response to calcium cytotoxicity, structural damage, and oxidative stress [35,47]. On the other hand, the aged retina is characterized by increased levels of reactive oxygen species (ROS), impaired autophagy, excessive energy consumption, and DNA damage, all of which contribute to the degeneration of RPE cells and link to AMD pathogenesis [48,49]. Besides, autophagy is not only linked to AMD but also to glaucoma, since dysregulation of autophagy can harm the outflow path of the trabecular meshwork, whereas promotion of autophagy via rapamycin treatment may have a cytoprotective effect upon oxidative stress [50].

Overall, an impairment of autophagy due to oxidative stress is related with several age-related eye diseases such as dry eye diseases, corneal dystrophy type 2, cataracts and retinal dystrophies like AMD, glaucoma, DR, and retinal artery occlusion [51,52].

### 6.2. Mitophagy and Oxidative Stress

Mitochondria are essential organelles that supply energy to the cell through oxidative phosphorylation (OXPHOS) and are also essential in calcium buffering, cell cycle control and regulation of apoptosis. Mitochondrial activity generates 1–5% ROS in physiological conditions [53]. Several studies demonstrated that autophagy may also display unique functions in axons, even independently from the function in soma and dendrites. In mammalian neurons, axons are rich in mitochondria, where ROS are predominantly produced. The acute burst of ROS in mitochondria may be a crucial activator of mitophagy, which is a process of mitochondria-selective autophagy in response to specific signals, including oxidative stress, starvation, and modification of mitochondrial proteins [54]. Mitochondrial dysfunction is an important pathological element mediating the onset of neurodegenerative disorders (e.g., *PARK2* mutations are associated with increased ROS and mitochondrial dysfunction in patients with Parkinson disease), and previous work in mouse models suggests that *Park2*-deficient photoreceptors exhibit ectopic mitochondria localization upon light exposure, thus suggesting that aberrant trafficking of damaged mitochondria might be one of the mechanistic aspects of retinal degeneration [55].

Under stress, a delicate balance between both mitochondrial fission and fusion is required to maintain functional mitochondria [53]. An excess of mitochondrial fission leads to mitochondrial fragmentation, whereas increased fusion results in mitochondrial elongation. Failure in any of the components of the machinery controlling mitochondrial dynamics may lead to RPE degeneration. Impairment of autophagy/mitophagy leads to increased protein aggregation in RPE and mitochondrial dysfunction evokes chronic RPE-derived inflammation [56].

Mitophagy is also relevant for the homeostasis of neuronal long axons subject to ROS and other stress insults. Retinal ganglion cells, whose long axons confluence to form the optic nerve, are particularly dependent on appropriate mitochondrial trafficking and dynamics in excess of ROS production by damaged mitochondria [57,58].

### 6.3. Cross-Talk between Autophagy and the Endoplasmic Reticulum (ER)-Mitochondria Axis in Oxidative Stress

ER–mitochondria interactions play a key role in the executing and regulating mitochondrial mitochondrial fission and fusion. For instance, in mitochondrial fission, the physical interaction between the membranes of the two organelles is directly demonstrated by the role of the ER tubules in encircling mitochondria and marking the sites of mitochondrial division. Thus, the ER regulates mitochondrial dynamics, and alterations in mitochondrial morphology uniquely reflect cell health. In non-oxidative conditions, the NLRP3 protein, a sensor of OS that triggers the formation of inflammasomes, is located on the ER. However, upon oxidative stress and in response to inflammation, NLRP3 relocates to ER–mitochondria contact sites (MAMs) thus enabling efficient sensing and monitoring of damaged mitochondria, which generate high levels of ROS. The ER and mitochondria reciprocally transmit danger signals through physical contacts, thus reinforcing their communications and triggering multiple and synergistic responses to oxidative stress, among them, promotion of autophagosome formation to degrade damaged mitochondria [59].

To sum up, autophagy plays a protective role against oxidative stress and other cell injuries but the accumulation of autophagosomes due to prolonged insult is also harmful to cells [60]. Therefore, the increase of autophagy in early stages of a disease or a punctual damaging input protects retinal cells, but sustained or dysfunctional autophagy may result harmful, particularly to photoreceptor and retinal ganglion cells. Indeed, dysfunctional autophagy, and particularly mitophagy, have been associated to diabetic retinopathy and glaucoma [61].

## 7. Oxidative Stress Effect on Retinal Lipid Peroxidation

The retina presents a high content of lipid composition. For this reason, alterations in lipids composition or balance may affect visual capacity. For instance, mutations in *ELOVL4* cause dominant Stargardt disease. ELOVL4 is an elongase that generates very long chain polyunsaturated fatty acids VLC-PUFA in the retina. Mutations in this gene have pleiotropic effects by directly altering VLC-PUFA signaling, enlarging the membrane rim curvature of photoreceptor disks and impacting on the size of ribbon synapses [62].

Lipid peroxidation is a consequence of ROS damage, and polyunsaturated fatty acids (PUFAs) are particularly susceptible to ROS. The high content of lipids in the retina and the constant exposure to light make this organ in special risk for lipid peroxidation. Once initiated by any of several pathways, lipid peroxidation, oxidative damage of membrane lipids, spreads aggressively in a self-propagating chain reaction, amplifying oxidative damage [63]. Lipid peroxides is reported to be related with the progression of diabetic retinopathy and age-related macular degeneration [64].

On the other hand, several studies have shown that accumulation of specific lipids, called lipofuscin, in RPE cells generates reactive oxygen species through phototoxicity. The accumulation of lipofuscin and subsequent generation of ROS upon light damage is a well-known triggering factor of macular degeneration in AMD and Stargardt disease [65]. Recent transcriptomic studies on RPE cells treated with oxidizing agents produced in the visual cycle showed differential expression of genes involved in oxidative stress response, angiogenesis, apoptosis, autophagy, and extracellular matrix remodeling, but particularly alteration of miRNAs and target genes as well as of extensive alternative splicing events that could also relate to the regulation of specific survival pathways [66,67].

## 8. Oxidative Stress Induces DNA Damage and Mutations

As aforementioned, oxidative stress induces a variety of structural and functional changes to lipids, proteins, and both nuclear and mitochondrial DNA. For instance, oxidative stress provokes accelerated telomere shortening. Telomeres are specialized structures at the end of chromosomes that contain characteristic repetitive G-rich DNA sequences (TTAGGG) that when damaged or shortened can induce an altered cellular phenotype that promote senescent traits. Some studies show that oxidative stress induces single-stranded breaks in telomeric DNA in RPE cells in vitro and cells with longer replicative life spans, which are more susceptible to oxidative stress and accumulate DNA damage [68].

Moreover, mtDNA is particularly sensitive to oxidative injury because: (i) mtDNA is localized close to the source of ROS production, (ii) it is not covered by histones, (iii) it is a circular intron-less circular DNA with high transcription rate, and (iv) the DNA repair system within the mitochondria appears to be less effective than that in the nucleus. mtDNA damage is particularly detrimental to non-dividing cells such as those in brain, heart skeletal muscles, photoreceptor and other retinal cells, such as and RPE cells [69]. Polymerase γ, which function is DNA repair, upon oxidative stress conditions it is oxidized causing a decreased activity. Consequently, photoreceptors are damaged and may favor the progression of retinal dystrophies such as AMD [70]. In response to oxidative damage, several cytoprotective pathways can be activated in RPE cells, e.g., via NFR2 and PGC1a, to promote mitochondrial biogenesis and mtDNA replication as well as to maintain telomere length [71]. In fact, an exquisite balance between mitochondria biogenesis and damaged mitochondrial clearance by either the ubiquitin-proteasome system and lysosomal mitophagy, and between mtDNA replication and repair pathways is crucial for proper RPE function. Any alteration of mitochondrial homeostasis leads to macular damage, a hallmark of AMD degeneration (as recently and comprehensively reviewed [56].

On the other hand, rare genetic mutations can cause specific sensitivity to oxidative and light stress in the retina. At least in mice, haploinsufficiency of *Mef2d* renders photoreceptors more susceptible to light-induced damage because they are unable to up-regulate *Nrf2* upon oxidative stress [72]. Besides, and at least in vitro, *SEMA4A* mutations make RPE cells more susceptible to light irradiation, ROS and ER stress [73].

Further work is required to identify polymorphic variants in relevant oxidative stress response genes that increase genetic susceptibility to oxidative stress in the retina, and may act as triggering factors in prevalent retinal degeneration pathologies (such as AMD and glaucoma) or as modifier genes in rare mendelian retinal disorders (such as RP, macular dystrophies, or Leber Hereditary Optic Neuropathy (LHON)).

## 9. Oxidative Stress, Genetic Factors, and Prevalent Retinopathies

### 9.1. Oxidative Stress and Glaucoma

Oxidative stress (OS) damages the DNA of the trabecular meshwork (TM), which is responsible for aqueous humor draining of the anterior eye chamber [74]. In glaucoma patients the levels of NOS and nitrosine in the TM are increased, thus reflecting high OS in the TM, which in turn lead to chronic changes in the aqueous and vitreous humor. Glaucoma phenotypic signature is the optic nerve retinopathy, due to the loss of retinal ganglion cells (RGCs). The apoptosis of RGCs is a direct consequence of axonal damage caused by high intraocular pressure, but also it is the indirect result of the increase in ROS caused by the death of RGCs affected by the ocular pressure. Therefore, intraocular pressure is the main factor causing ganglion cell death, although progression of glaucoma is also dependent on reduced optic disk perfusion, which result in the subsequent activation of hypoxia response genes [74].

*Optineurin* (*OPTN*) is a glaucoma-causative gene. Perfusion studies show that *OPTN* expression increases after 2 to 7 days of elevated IOPs (intraocular pressure) [75]. OPTN has been also proposed to exert a protective role in glaucoma development [76]; however, in a transgenic mouse model that overexpressed *Optineurin*, high levels of this protein in the lens and the retina failed to protect against transforming growth factor-β1–induced apoptosis [77]. Other investigators showed that *Optineurin* overexpression protects cells from hydrogen peroxide-induced cell death, and the OPTN E50K mutation decreased the neuroprotective effect by compromising mitochondrial membrane integrity, which resulted in cells less fit to survive under stress conditions [78]. Indeed, the role of *Optineurin* in glaucoma pathogenesis requires further study, particularly, whether its overexpression can be a protective measure in retina upon oxidative stress.

### 9.2. Oxidative Stress and Age-Related Macular Degeneration (AMD)

AMD is one of the main causes of blindness in western countries, characterized by the loss of RPE cells and photoreceptors in the macula area. AMD retinas display extracellular deposits, known as druses, between Bruch’s membrane and the RPE [79]. RPE cells are prone to produce ROS due to the high metabolic rate. Moreover, these cells are exposed to ROS as a consequence to iron ions accumulation, sunlight exposure, or cigarette smoke. The macular pigment, composed by two dihidroxycarotenoids (lutein and zexantin), is a natural barrier that protects the macula from OS. Advanced glycation end-products (AGEs) and carboxyethylpyrrole adducts produced by fatty acids oxidation in the photoreceptor tips have been detected in drusen isolated from AMD samples [79].

Usually, RPE damage is observed in early stages of AMD although there is not a direct link between the extent of damage in the RPE caused by OS and AMD progression [80]. RPE-damaged cells release cytokines and chemokines that recruit and activate choroid dendritic cells, which amplify the inflammatory process due to cell–cell contact, overall triggering a chronic inflammation that has been reported to play a key role in the AMD pathogenicity [81]. Indeed, genetic risk variants may exacerbate the inflammatory response due to OS in the retina, and several polymorphic variants in genes encoding or regulating key complement proteins have been associated to AMD [82]. The major genetic risk factor to develop AMD is the Y402H polymorphism of complement factor H (CFH), which reduces CFH’s ability to neutralize the effect of oxidized photoreceptor phospholipids from photoreceptors, which are incorporated into the RPE membrane and trigger RPE cell apoptosis [83,84]. However, other genetic and external stimuli must contribute to the initiation of the complement cascade and inflammatory response. For instance, single nucleotide polymorphisms (SNPs) in complement factor genes (including *CFH*) account for ∼46–71% of the genetic risk for developing AMD. Concerning external triggers, smoking—and consequently, oxidative stress—is the largest modifiable external risk factor for AMD. Genotype and smoking have been independently related to AMD with synergic effects [85]. Recently, TLR2 has been also reported as a mediator between oxidative stress damage in the RPE and the development of complement-mediated retinal pathology in AMD and other retinal neurodegeneration pathologies, so that TLR2 blockade protects photoreceptors and RPE from oxidative stress-induced cell death [86].

On the other hand, autophagy is a protective homeostatic mechanism designed to remove cellular components including those damaged by oxidative damage. Compromised autophagy contributes in AMD as a result of the RPE dysfunction [87]. Additionally, RPE has antioxidant systems that protect cells against oxidative damage. Several studies identified the NRF2 axis as an essential signaling system in the RPE. In addition to its antioxidant protection, the NRF2-signaling pathway is involved in maintaining mitochondria metabolism by controlling the expression of several Krebs cycle enzymes, which increases glucose flux to generate NADH, an essential substrate for electron transport chain complex I to promote ATP production [88,89]. Chronic cigarette smoking impairs NRF2 signaling in the RPE, which is associated with RPE degeneration, but not AMD progression [90,91,92]. In addition to behavioral factors such as cigarette smoking and high fat diet ingestion, photo-oxidative stress and increased phagocytosis of photoreceptor outer segments most probably induce further dysfunction of the RPE and increase photoreceptor cell death during advanced stages of dry non-neovascular AMD [93].

### 9.3. Oxidative Stress and Diabetic Retinopathy (DR)

DR is one of the most common Diabetes *mellitus* complications. Molecular mechanisms of high glucose levels causing blindness are yet to be completely determined, although chronic hyperglycemia lead to several biochemical changes, among them, the activation of protein kinase C, polyols accumulation through aldose reductase pathway, AGEs production increment and free radical overproduction. These free radicals are generated from glucose auto-oxidation reactions and glycated adducts of proteins. These metabolic changes increase the levels of pro-inflammatory cytokines, chemokines and other mediators of the inflammatory response stimulating an influx of leucocytes and altering vascular permeability, which altogether favors the degeneration of the retina [21,26,61,94].

## 10. Oxidative Stress, Genetics, and Inherited Rare Diseases of the Retina

### 10.1. Oxidative Stress and Retinitis Pigmentosa (RP)

RP, caused by mutations in many different genes, is the most prevalent among rare inherited retinal disorders (IRDs) (prevalence of 1:4000). The main phenotypic feature of RP is the progressive loss of rods followed by the death of cones. During the last years, OS has been shown to play a relevant role in RP pathogenesis and disease progression. Depending on the RP causative gene, antioxidant treatments might preserve cone function and prolong rod survival. Several studies show that both endogenous OS produced during retinal metabolism, such as lipid peroxidation or DNA damage, and exogenous OS, such as those produced by sunlight exposition, contribute to photoreceptor cell death. Moreover, rod photoreceptor death cause that higher levels of oxygen permeate into the tissue, thus increasing ROS production and inducing cell oxidative damage into the surviving cells (mostly cones), which will eventually result in cone death [17,95,96]. Since the retina is a highly-metabolic demanding tissue, retinal cells have developed protective mechanisms against damaging agents, among them, autophagy activation. In retinal ganglion cells (RGCs), autophagy exerts a clear cytoprotective role by minimizing ROS levels and sustaining mitochondrial function [57,97].

Indeed, some RP-causative mutations cause misfolding of highly expressed transmembrane proteins that are involved in photoreception or phototransduction (such as rhodopsin, phosphodiesterase 6 subunits, or arrestin). The accumulation of unfolded or misfolded proteins cause UPR and an increase in oxidative stress in the ER, and elicits a compensative transcriptional program, for instance through PERK and IRE1 pathways, to activate genes encoding antioxidant enzymes. Chronic IRE1 activation causes an increase or mitochondrial ROS in degenerating retinas [98].

Some studies suggest that autophagy could have beneficial effects in RP while others indicate it could negatively affect photoreceptor death. This apparent contradiction may be reconciled if we consider that: (i) RP is a highly heterogenous genetic disease, with many different genes and mutations associated to this disorder, therefore the importance of autophagy in retinal cells might be different depending on the mutation; (ii) autophagy may play different roles in the death of rods and cones; and finally (iii) moderate levels of autophagy in the photoreceptors may be beneficial whereas excessive autophagy may be deleterious [35]. Again, a delicate balance between autophagy promotion or reduction depending on the cell requirements, the intensity and time duration of the stress stimulus may be the clue to understand the relevance of this pathway in retinal homeostasis, particularly when retinal cells are already under distress by genetic mutations.

To keep oxidation within controllable levels, cells have to activate and deploy endogenous antioxidant defenses. Inactivating mutations or expression deficiency of genes encoding antioxidant enzymes may lead to a faster progression of the disease. Glyoxalase 1 (GLO 1) is a ubiquitous cellular enzyme involved in detoxification of methylglyoxal (MGO), a cytotoxic byproduct of glycolysis. MGO excess can cause oxidative stress through the inactivation of antioxidant detoxification enzymes such as glutathione peroxidase and SOD. Hypomorphic genetic variants associated to diminished *GLO1* expression have been identified in a Sicilian population. As a result, retinal cells are more sensitive to oxidative stress-damage and eventually die by apoptosis, thus leading to retinal degeneration [99].

In fact, mutations in many different genes involved in a large variety of functions cause mendelian inherited retinal dystrophies (IRDs). Among all the IRD causative genes, only *CERKL* has been implicated as a resilience gene against oxidative stress [100]. The precise function of *CERKL* is yet to be determined but several studies show that it is involved in the cellular response to oxidative stress and may play a role in protecting cells against stress injury [101,102]. Besides, CERKL shows a highly dynamic subcellular localization, as it can shuttle from nucleus to cytoplasm, it is also associated to the Golgi and Trans-Golgi vesicles, endoplasmic reticulum, and mitochondria membranes [100,101,102]. *CERKL* is not only expressed in the retina but also in several adult and fetal tissues in human and mouse. In humans, the highest expression of *CERKL* is detected in the retina, some brain regions, lung, and kidney [103], while in adult mice, the highest levels of expression can be detected in the retina, liver, and testis [104]. Mutations in *CERKL* have been reported to cause RP and cone-rod dystrophy (CRD), thus providing further support to the concept that failure in the endogenous resilience mechanisms to overcome oxidative stress (for instance, by gene mutations) leads to an accelerated progression of retinal neurodegeneration.

The high genetic heterogeneity of RP not only lies on gene mutation but most probably on gene regulation and ability of photoreceptors and RPE to respond to external toxic stimuli and metabolic challenges as well. In this respect, miRNAS and lncRNAs may regulate relevant IRD-causative genes in response to oxidative stress injuries, as reported for miRNAs (hsa-miR-1307, hsa-miR-3064, hsa-miR-4709, hsa-miR-3615 and hsa-miR-637), in RPE cells challenged with oxidizing compounds. At least, five RP causative genes (*KLHL7*, *RDH11*, *CERKL*, *AIPL1*, and *USH1G*) emerged as validated targets, thus suggesting a tight connection between induced oxidative stress and RP onset and progression [66].

### 10.2. Oxidative Stress and Leber Hereditary Optic Neuropathy (LHON)

LHON is a clear example of retinopathy associated to oxidative stress. This rare disease is characterized by the degeneration of RGCs due to mutations in the mitochondrial genes encoding for the NADH dehydrogenase, an enzyme from the oxidative phosphorylation chain of the mitochondria. Mutations in the genes *MT-ND1*, *MT-ND4*, *MT-ND4L*, and *MT-ND6* cause a decrease in ATP synthesis and a high increase of intracellular ROS levels. The mitochondrial electron transport disruption leads to an increment of ROS, thus interfering with glutamate transport and triggering apoptosis of the RGCs [74,105].

## 11. Potential Therapies for Retinal Diseases in front of Oxidative Stress

ROS underlie the pathophysiology of diverse neurodegenerative diseases. In the retina, the source and impact of ROS are different depending on the pathology. Finding new therapies to avoid damaging ROS formation will be a step forward on preventing or slowing down the progression of these highly incapacitating blinding diseases, whether prevalent or rare. The strategies could be either to enhance the production of antioxidant enzymes, to reduce ROS, or to promote cytoprotective signaling pathways.

As a complementary therapy, nutritional changes and diet supplementation with antioxidant properties could have protective effects on ROS produced in the progression of some retinal diseases, depending on the altered signaling pathway [94]. A list of reported treatments can be found in Table 1.

Another therapeutic approach is targeting ROS, directly or indirectly, with small molecules or drugs [18], as summarized in Table 2.

Finally, other treatments for retinal dystrophies are based on promoting cytoprotective pathways. As aforementioned, RP is a mendelian rare disease caused by mutation in many genes but increasing evidence in patients and animal models suggests that oxidative stress and inflammation contribute to its pathogenesis irrespective of the causative gene. In fact, sustained chronic inflammation including elevated TNFα and other interleukines have been reported in the aqueous humor of patients and in mouse animal models of RP, as well as in AMD, glaucoma, and DR [139,140]. Inhibition of inflammatory pathways by TNFα-blockade using anti-TNF antibodies (Infliximab) was explored as a potential avenue of treatment on porcine retinal explants. A clear neuroprotective effect of TNFα blockers, with a reduction of oxidative stress markers and a decrease of both reactive gliosis and apoptosis, was observed in retinal organotypic cultures.

Other authors have resorted to TLR2 blockade. TLR2 induces complement factors in both retinal pigment epithelial (RPE) cells and mononuclear phagocytes. Neutralization of TLR2 reduces complement deposition, microglial activation, and protects photoreceptor neurons from oxidative stress-induced degeneration. TLR2 deficiency also promotes RPE resilience [86].

On the other hand, modulation of other cell cytoprotective mechanisms, such as autophagy, including specific organelle-phagy [57] and mitochondrial dynamics [53], or the regulation of SG generation in the retina [141], may provide potential cytoprotective strategies for IRD treatment.

The most interesting conclusions of this type of work is that therapeutic strategies to reduce oxidative stress and inflammation (by targeting TNFα, TLR2, or other signaling cell pathways) are promising approaches, which should be concurrently explored as amelioration treatments of symptoms for a wide cohort of patients in addition to gene therapy, which is currently based on the correction of genetic defects caused by mutations and is thus restricted to a handful of patients.

## 12. Conclusions

Oxidative stress by exogenous or endogenous agents damage the retina and the RPE, triggering apoptosis of photoreceptors and retinal ganglion cells. In prevalent diseases, such as AMD, glaucoma, and diabetic retinopathy, oxidative stress is a trigger of the pathologies, worsening the physiology of retinal cells and accelerating the progression of the disease. In rare genetic disorders, mutations in several causative genes cause metabolic distress and decrease cell resilience to oxidative stress, adding to the progressive attrition of photoreceptors and RGCs, eventually leading to blindness. We hypothesize that other IRD causative genes related to oxidative stress sensing and oxidative stress management, such as genes implicated in the control of autophagy, stress granule formation, or mitochondrial dynamics, will be identified in the near future, widening the landscape of genes and mutations that cause visual disorders. In addition to the more conventional gene therapy tailored to treat specific genetic defects, treatments addressed to increase cytoprotective signaling pathways in front of oxidative stress and to promote retinal cell resilience will provide new avenues to halt the progression of these blinding diseases and restore retinal homeostasis.

## Figures and Tables

**Table 1:** Dietary supplementation with antioxidant compounds in retinal dystrophies.

Agent	Function	Retinal Dystrophy
Xanthophylls	Useful in protecting the retina from OS and ameliorating oxidative stress states	DR, AMD and RP [106,107,108,109]
Zinc and Manganese	Zinc supplements exhibit antioxidant and anti-inflammatory activities, delaying oxidative processes in the long term, and manganese is a cofactor of many antioxidant agents as SODs.	DR and AMD (https://clinicaltrials.gov/) [110,111,112]
Alpha-lipoic acid	Essential for mitochondrial function and also induces NRF2 binding to antioxidant response elements. which is important to slow down	DR, glaucoma, AMD, RP [113,114,115]
Curcumin	Increases the transcription of antioxidant enzymes and induces the activation of NRF2 helping to decrease oxidative stress in the retina.	Glaucoma, DR and AMD [116,117]
Ubiquinone or coenzyme Q10	Inhibits the formation of free radicals reducing the effects of oxidative stress. Moreover, it activates eNOS and mitochondrial OXPHOS decreasing blood pressure.	AMD, RP, DR and glaucoma [118]
Omega 3	Deficient consumption of omega-3 contributes to the degeneration of the retina. Additionally, PREDIMED (Prevention with Mediterranean Diet) is a clinical trial that followed for 6 years individuals with diabetes mellitus type 2. Patients whose diet included omega-3 polyunsaturated fatty acids, showed a 48% decreased incidence in DR.	Glaucoma, RP and DR [119,120,121,122]
Spermidine	Acts as a ROS scavenger by sequestering singlet oxygen and reactive species and reduced RGC death.	Glaucoma [123]
Lycopene	Decreases NF-κB activation and ROS production in RPE cells, while increasing Nrf2 and GSH levels.	AMD [124]
Resveratrol	Shows antiproliferative, antiangiogenic, antioxidant, endothelial, anti-inflammatory, antiplatelet, and neurogenic activity.	Glaucoma, cataract, AMD and DR [125]

**Table 2:** Drugs and compounds that reduce reactive oxygen species (ROS) production in retinal cells.

Agent	Function	Retinal Dystrophy
Selective siRNAs (SYL040012)	Target specific β-receptors and have been demonstrated to decrease by 50% intraocular pressure 4 days after ocular administration, thus concomitantly reducing oxidative stress in RGCs.	Glaucoma [126]
17β-estradiol	Inhibits ROS production, preserves ATP production and mitochondrial membrane potential, and decreases cellular and mitochondrial calcium loading. Moreover, estrogen’s neuroprotective effects have been tested in RGCs in an in vivo model of glaucoma	Glaucoma [127,128]
Celastrol	Intravitreal injection reduces IOP protecting RGC from death by activating the cellular antioxidant defense system, attenuating of microglial activation, inhibiting of tumor necrosis factor (TNF)-alpha, and nitric oxide synthase production.	Glaucoma [129]
SERPINA3K	Intravitreal injection of this serine protease inhibitor demonstrated to block the expression of VEGF and TNF-α. Moreover, it suppresses ROS production and upregulate manganese superoxide dismutase and glutathione levels.	DR [130]
PHA666859	Blocks the p38 MAPK pathway, which plays a role in inflammatory processes.	DR [131]
E3330	Exhibits RPE-protective effects downregulating intracellular ROS by attenuating the levels of NF-κB as well as the secretion of monocyte chemoattractant protein-1.	AMD [132]
BSIH	Iron favors ROS formation in the retina, for this reason, BSIH which is a protochelator is a potential therapeutic molecule. BSIH involves iron sequestration that occurs only when the cells are stressed by hydrogen peroxide.	AMD [133]
NAC (*N*-acetyl-l-cysteine)	Inhibits focal adhesion kinase, a kinase whose phosphorylation, induced by ROS, cause apoptosis.	DR [134,135]
Ranibizumab and aflibercept	Present a therapeutic effect against acrolein-induced oxidative cytotoxicity in human ARPE-19 cells via an increase in mitochondrial bioenergetics.	AMD [136]
AAV-SOD2	gene therapy by overexpression of SOD2 introduced by adeno-associated viruses (AAV-SOD2) is able to attenuate oxidative stress and improve mitochondrial dysfunction of RGC and optic nerve secondary to glaucoma.	Glaucoma [137]
Retinoids	Systemically administration reduces retinal OS and correct rod dysfunction.	DR [138]
